# Altered small dense LDL profiles in long-standing controlled type 1 diabetes

**DOI:** 10.3389/fendo.2026.1804987

**Published:** 2026-04-07

**Authors:** Helena Sardà, Arnau Solé, Cristina Colom, Carla Borràs, Gemma Carreras, Sonia Benítez, Inka Miñambres, Joan Carles Escolà-Gil, José Luis Sánchez-Quesada, Antonio Pérez

**Affiliations:** 1 Department of Endocrinology and Nutrition, Hospital de la Santa Creu i Sant Pau – Hospital Dos de Maig, Barcelona, Spain; 2 Department of Medicine, Universitat Autònoma de Barcelona, Bellaterra, Spain; 3 Pathophysiology of Lipid Related Diseases, Institut de Recerca Sant Pau (IR-Sant Pau), Barcelona, Spain; 4 Department of Biochemistry and Molecular Biology, Universitat Autònoma de Barcelona, Bellaterra, Spain; 5 Centro de Investigación Biomédica en Red de Diabetes y Enfermedades Metabólicas (CIBERDEM), Madrid, Spain; 6 Department of Pediatrics, Hospital de la Santa Creu i Sant Pau, Barcelona, Spain; 7 Department of Pediatrics, Obstetrics and Gynecology, and Preventive Medicine and Public Health, Universitat Autònoma de Barcelona, Bellaterra, Spain; 8 Institut de Recerca Sant Pau (IR-Sant Pau), Barcelona, Spain; 9 Cardiovascular Biochemistry, Institut de Recerca Sant Pau (IR-Sant Pau), Barcelona, Spain

**Keywords:** apolipoprotein C3, cardiovascular disease risk, cholesteryl ester transfer protein, direct assay, hepatic lipase, small dense LDL cholesterol, type 1 diabetes

## Abstract

**Introduction:**

Small dense low-density lipoprotein (sdLDL) is a highly atherogenic LDL subclass associated with cardiovascular disease (CVD). While type 1 diabetes confers increased cardiovascular risk despite adequate glycemic control, the role of sdLDL and its regulators remains unclear.

**Methods:**

In this cross-sectional observational study, plasma from 69 individuals with long-standing type 1 diabetes and 24 healthy controls was analyzed. sdLDL-cholesterol (sdLDL-C) concentration, sdLDL-C/LDL-cholesterol ratio, LDL size and subclasses were assessed using homogeneous assays, NMR spectroscopy, and gradient gel electrophoresis. Apolipoprotein C3 (ApoC3), hepatic lipase (HL), endothelial lipase (EL), and cholesteryl ester transfer protein (CETP) activity were measured by immunoturbidimetric, ELISA and functional assays.

**Results:**

Despite adequate glycemic control (mean HbA1c 7.6% [60 mmol/mol]) and near-normal lipid levels, individuals with type 1 diabetes had significantly higher sdLDL-C (0.56 ± 0.28 mmol/L vs 0.43 ± 0.26 mmol/L), increased sdLDL-C/LDL-cholesterol ratio (0.20 ± 0.08 vs 0.12 ± 0.06) and smaller LDL particle size (26.32 ± 1.08 nm vs 26.81 ± 0.68 nm) compared with controls. ApoC3 and HL mass/activity were significantly increased (8.67 ± 3.22 mg/dL vs 6.53 ± 2.42; 46.60 ± 16.12 ng/mL vs 15.45 ± 7.40 ng/mL and 1.03 ± 0.24 U/mL vs 0.89 ± 0.23 U/mL; respectively), CETP activity significantly reduced (808.8 ± 197.0 pmol/mL/h vs 929.7 ± 149.6 pmol/mL/h), and endothelial lipase levels unchanged. sdLDL-C positively correlated with ApoC3 (r = 0.7517) and inversely with CETP activity (r = -0.2682).

**Conclusion:**

Long-standing type 1 diabetes with adequate glycemic control is associated with an atherogenic sdLDL profile despite near-normal conventional lipid levels. This first multi-method characterization study of sdLDL in type 1 diabetes highlights the contribution of ApoC3, CETP and HL to sdLDL-C enrichment and suggests that direct assessment of sdLDL may improve cardiovascular risk stratification.

## 1 Introduction

Individuals with type 1 diabetes have a significantly increased risk of cardiovascular disease (CVD) compared with the general population (1). Although glycemic control remains a key determinant of long-term outcomes, individuals with apparently good glycemic control continue to face an elevated risk of cardiovascular events, even in the absence of multiple additional risk factors (2). Thus, traditional cardiovascular risk factors cannot fully explain this process, and the underlying molecular mechanisms remain incompletely understood.

Over the past decades, growing attention has been directed toward the role of lipoproteins in type 1 diabetes, as quantitative, qualitative, and functional abnormalities have all been proposed to contribute to excess cardiovascular risk (3–6). In type 2 diabetes, dyslipidemia is a major driver of CVD and typically includes elevated triglycerides (TG), reduced high-density lipoprotein cholesterol (HDL-C), and the predominance of small dense low-density lipoprotein (sdLDL) particles (7). By contrast, conventional lipid profiles in type 1 diabetes are often reported as similar to, or even less atherogenic than those in the general population (3, 4). This suggests that qualitative rather than quantitative lipoprotein alterations may play a particularly important role in type 1 diabetes.

Among these qualitative disturbances, low-density lipoprotein (LDL) particle size is of special relevance. sdLDL particles are highly atherogenic because of their prolonged plasma residence, increased susceptibility to glycation and oxidation, reduced affinity for the LDL receptor, and enhanced arterial wall retention (3). While sdLDL are typically associated with insulin resistance and hypertriglyceridemia (8), their presence in type 1 diabetes is somewhat unexpected given the usually normal TG and HDL-C levels. Nevertheless, prior studies have reported conflicting results, likely reflecting both differences in patient characteristics and methodological approaches (5, 9–12).

The SEARCH study (5) demonstrated that youth patients (age between 10–22 years) with type 1 diabetes, irrespective of glycemic control, had higher sdLDL levels than their peers without diabetes when measured by ultracentrifugation. Other studies have similarly reported increased sdLDL in women and adolescents with type 1 diabetes using size exclusion chromatography (10, 13). However, studies using technologies more easily applicable to clinical studies, such as gel electrophoresis (GGE) or nuclear magnetic resonance (NMR) spectroscopy, have shown divergent findings, ranging from decreased to increased LDL size (9, 11, 14, 15). Comparative analyses indicate only moderate agreement between GGE and NMR, precluding a direct comparison between both methods (16). More recently, a direct homogeneous assay for sdLDL-cholesterol (sdLDL-C) has been developed (17), with large Japanese cohorts demonstrating its superiority over conventional lipid fractions in predicting CVD risk (18). This underscores the value of applying complementary methods to characterize sdLDL-C in type 1 diabetes.

The mechanisms underlying sdLDL-C elevation in type 1 diabetes, particularly in the absence of insulin resistance and hypertriglyceridemia, remain poorly understood. Disturbances in lipid regulatory proteins —including apolipoprotein C3 (ApoC3), hepatic lipase (HL), cholesteryl ester transfer protein (CETP) activity— have been described in type 1 diabetes and may promote sdLDL-C formation independently of conventional lipid levels (19–24).

The aim of the present study was to evaluate sdLDL-C levels in individuals with type 1 diabetes compared with healthy controls (HC) using multiple complementary methodologies, and to investigate potential alterations in proteins involved in the maturation of LDL, such as ApoC3, HL, CETP and endothelial lipase (EL), which may contribute to sdLDL-C formation.

## 2 Materials and methods

This was a cross-sectional observational study including 69 Caucasian patients with type 1 diabetes routinely followed at a tertiary university hospital since their diagnosis between 1985 and 1994 (25). The diagnosis of type 1 diabetes was established according to international guidelines (26). No predefined exclusion criteria were applied, and all eligible patients were invited to participate; the final sample consisted of those who agreed to participate and attended the study visit during the recruitment period. All the subjects provided written informed consent to participate. The protocol was approved by the clinical research ethics committee of the Hospital de la Santa Creu i Sant Pau (IIBSP-DIA-2011-114). Twenty-four HC normolipidemic and normoglycemic, with no personal or family history of premature coronary artery disease, major cardiovascular risk factors, or known infectious or inflammatory conditions were included as control group. All procedures performed involving human participants were in accordance with the 1964 Helsinki declaration and its later amendments or comparable ethical standards.

The following baseline characteristics were recorded: sex, age, weight, height, body mass index (BMI), time since diagnosis, dose of insulin expressed as units per kg per day (UI/kg/day), smoking status and the presence of comorbidities or diabetes-related chronic complications. Dyslipidemia was defined as the presence of any of the following: TG ≥ 1.7 mmol/L, LDL-cholesterol (LDL-C) > 4.2 mmol/L or lipid lowering therapy. Hypertension was defined as the presence of three or more systolic blood pressure measurements ≥ 140 mmHg and/or diastolic blood pressure measurements ≥ 90 mmHg, or antihypertensive treatment.

The biochemical parameters analyzed included total cholesterol (TC), TG, HDL-C, LDL-C, very low-density lipoprotein cholesterol (VLDL-C), apolipoprotein B (ApoB) and glycosylated hemoglobin A1c (HbA_1c_), as previously described (25).

sdLDL-C was quantified with a homogeneous assay (Denka Co., Ltd., Tokyo, Japan), also adapted for the Cobas 6000/501c platform (Roche Diagnostics, Basel, Switzerland). In the first step, triglyceride-rich lipoproteins, high-density lipoprotein (HDL), and large buoyant LDL were dissociated by a polyoxyethylene benzylphenyl ether derivative and sphingomyelinase, while sdLDL particles were preserved by a polyoxyethylene styrenephenyl ether derivative. In the second step, a polyoxyethylene alkyl ether derivative selectively dissociated sdLDL, and the released cholesterol was measured enzymatically using cholesterol esterase and cholesterol oxidase (17). ApoC3 levels (Randox Laboratories, Crumlin, UK) were quantified by immunoturbidimetric assay adapted for the Cobas 6000/501c platform (Roche Diagnostics, Basel, Switzerland).

LDL particle size was determined by non-denaturing gradient gel electrophoresis (2–16%) according to the method of Nichols et al. (27), with modifications (28). Briefly, two solutions of acrylamide at 2% and 16% were prepared using a stock solution of acrylamide and bisacrylamide (30% total, 5% cross-linker) and mixed using two peristaltic pumps in a Mini Protean 3 Multicaster Cell Chamber (Bio Rad, Hercules, CA, USA). Fifteen µl of total plasma were preincubated for 15 min with 5 µl of Sudan Black (0.1% w/v in ethylene glycol), and 5 µl of sucrose (50% w/v). Fifteen µl of this mixture were electrophoresed at 4 °C for 30 min at 20 V, 30 min at 70 V, and 8 h at 100 V. Gels were scanned at 595 nm, and particle size was calculated using a plasma pool with four reference standards of LDL size, as described (28). Schematics diagrams illustrating the analytical methods used are included as Supplementary File (**Supplementary Figure 1**).

Plasma lipoprotein particle number was determined using the 2D ^1^H-NMR Liposcale test (Biosfer Teslab, Reus, Spain), a diffusion-ordered spectroscopy-based method that estimates lipoprotein size from diffusion coefficients using the Stokes–Einstein equation (29). Briefly, plasma samples were analyzed by ¹H-NMR spectroscopy at 600 MHz using a double stimulated echo pulse sequence with bipolar gradient pulses. The methyl region of the spectra was surface-fitted with nine functions corresponding to lipoprotein subclasses. Lipoprotein classes were assigned according to NMR-derived size ranges (VLDL, LDL, HDL). Cholesterol and TG concentrations of the main lipoprotein fractions were predicted using partial least squares regression models calibrated against reference ultracentrifugation-based measurements. Particle numbers were estimated by dividing lipid volume by particle volume, and subclass particle numbers were obtained from the relative spectral areas. The parameters that have been determined by this methodology are the following: VLDL-C (mg/dL), intermediate density lipoprotein cholesterol (IDL-C) (mg/dL), LDL-C (mg/dL), HDL-C (mg/dL), VLDL-TG (mg/dL), IDL-TG (mg/dL), LDL-TG (mg/dL), HDL-TG (mg/dL), VLDL-C/TG ratio, LDL-C/TG ratio, HDL-C/TG ratio, VLDL-P (nmol/L), Large VLDL-P (nmol/L), Medium VLDL-P (nmol/L), Small VLDL-P (nmol/L), LDL-P (nmol/L), Large LDL-P (nmol/L), Medium LDL-P (nmol/L), Small LDL-P (nmol/L), HDL-P (μmol/L), Large HDL-P (μmol/L), Medium HDL-P (μmol/L), Small HDL-P (μmol/L). C indicates cholesterol; TG indicates triglycerides; P indicates particles.

CETP activity was measured using a commercial assay kit (Sigma-Aldrich/Merck, St. Louis, MO, USA). This assay quantifies CETP-mediated transfer of a fluorescent neutral lipid from a donor substrate to a physiological acceptor, independent of endogenous lipoprotein concentrations in plasma.

HL concentrations were measured using a Human LIPC ELISA kit based on the sandwich ELISA principle (Ardent Bio LLC, Houston, TX, USA). Absorbance was read at 450 nm, and HL concentrations were calculated from a standard curve. EL concentrations were determined using a double antibody sandwich ELISA kit (Wuhan Fine Biotech Co., Wuhan, China). Optical density was measured at 450 nm, and EL concentrations were calculated from a standard curve. HL activities were measured using a commercial kit (Beijing Solarbio Science & Technology Co., Ltd., Beijing, China). The assay is based on the hydrolysis of α-naphthyl acetate by HL to generate α-naphthol, which reacts with fast blue B salt to form a purple-red azo compound with a characteristic absorption peak at 595 nm. Schematics diagrams illustrating the analytical methods used are included as Supplementary File (**Supplementary Figure 2**).

The statistical analyses were performed using the Statistical Package for the Social Sciences (SPSS, Inc., Chicago, IL, USA) version 29.0 for Windows and GraphPad Prism version 8.0.2 for Windows (GraphPad Software, San Diego, CA, USA). Variables are expressed as mean ± standard deviation (SD) for continuous variables with a normal distribution, as median and interquartile range (IQR) for continuous variables with a non-normal distribution, and as absolute numbers with percentages for categorical variables. We used the parametric chi-square test to compare categorical variables. Student’s t test was used to compare categorical and continuous variables with a normal distribution, and the Mann–Whitney U test was performed for nonparametric variables. The Spearman’s rho test was used to analyze the correlation between continuous variables. p < 0.05 was considered to indicate statistical significance.

## 3 Results

### 3.1 Baseline characteristics of study participants

The demographic, clinical and biochemical characteristics of patients with type 1 diabetes and HC are shown in **Table 1**. Compared with HC, patients with type 1 diabetes had lower TC, LDL-C and ApoB, while no significant differences were observed in HDL-C, TG and VLDL-C levels.

**Table 1:** Demographic, clinical and biochemical characteristics of patients with type 1 diabetes and healthy controls (HC).

Variables	HC (n = 24)	Type 1 diabetes (n = 69)	p
Sex M/F, *n* (%)	9 (37.5) / 15 (62.5)	40 (58) / 29 (42)	0.084
Age, years	42.92 ± 10.97	46.92 ± 7.01	0.139
Time since diagnose, years	–	22.42 ± 2.17	–
Insulin dose, Ui/Kg/day	–	0.63 ± 0.20	–
BMI, Kg/m^2^	24.63 ± 3.11	26.26 ± 3.55	0.215
Smoking status			
Smoker Former Smoker Non-Smoker	–	25 (36.2) 19 (27.5) 25 (36.2)	–
Dyslipidemia	–	36 (52.2)	
Lipid lowering therapy	–	31 (44.9)	–
Statin treatment	–	30 (43.5)	–
Statins and ezetimibe treatment 1	–	1 (1.4)	–
Hypertension	–	19 (27.5)	–
Diabetes-related retinopathy	–	11 (16.2)	–
Diabetes-related nephropathy	–	6 (8.7)	–
Diabetes-related neuropathy	–	11 (16.9)	–
CHD	–	1 (1.4)	–
Stroke	–	1 (1.4)	–
Peripheral arterial disease	–	2 (2.8)	–
HbA_1c_, mmol/ml	32.57 ± 3.25	59.50 ± 11.73	**< 0.001**
HbA_1c_, %	5.13 ± 0.29	7.59 ± 1.07	
Total cholesterol, mmol/L	5.35 ± 0.85	4.75 ± 0.71	**0.001**
HDL cholesterol, mmol/L	1.54 ± 0.35	1.48 ± 0.30	0.452
LDL cholesterol, mmol/L	3.37 ± 0.75	2.82 ± 0.54	**< 0.001**
VLDL cholesterol, mmol/L	0.43 (0.31-0.52)	0.37 (0.27-0.45)	0.178
Triglycerides, mmol/L	0.92 (0.67-1.12)	0.80 (0.59-0.97)	0.194
Apolipoprotein B, g/L	0.90 ± 0.19	0.77 ± 0.15	**0.002**
sdLDL-C, mmol/L	0.43 ± 0.26	0.56 ± 0.28	**0.0496**

M, Male; F, Female; BMI, Body Mass Index; CHD, Coronary Heart Disease. Data is expressed as mean ± SD, median (IQR) or n (%). Bold numbers indicate statistically significant differences.

### 3.2 LDL particle characteristics

**Figure 1:**
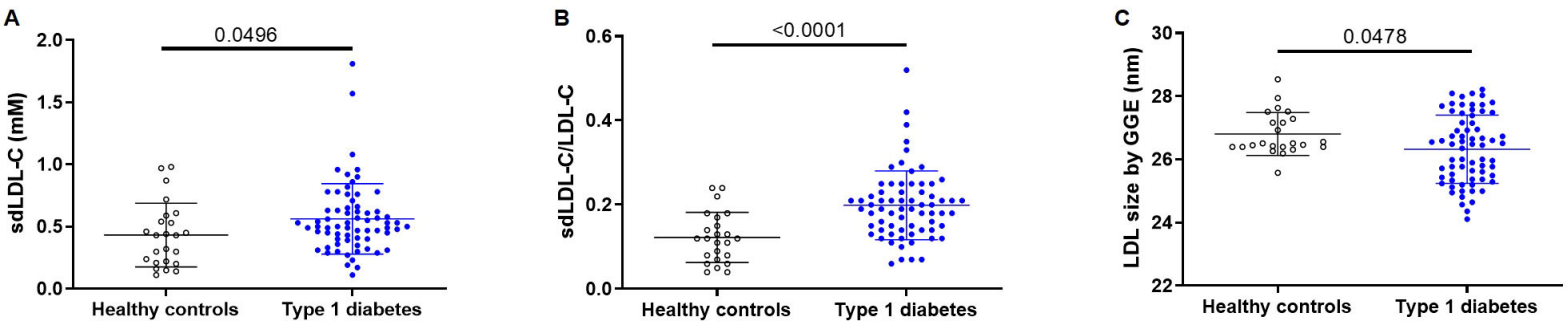
Title: sdLDL-C levels, sdLDL-C/LDL-C ratio, and LDL size compared between type 1 diabetes and healthy controls. **(A)** sdLDL-C measured by the commercial automated method. **(B)** sdLDL-C/LDL-C ratio **(C)** LDL size determined by non-denaturing polyacrylamide gradient gel electrophoresis. Horizontal lines indicate statistical differences.

In patients with type 1 diabetes, despite having reduced LDL-C levels, sdLDL-C concentration was elevated compared to HC (0.56 ± 0.28 mmol/L and 0.43 ± 0.26 mmol/L, respectively; p = 0.0496), and consequently, the sdLDL-C/LDL-C ratio was also increased (0.20 ± 0.08 vs 0.12 ± 0.06; p < 0.0001). LDL particle size measured by GGE was significantly reduced (26.32 ± 1.08 nm vs 26.81 ± 0.68 nm; p = 0.0478), which is in line with the increase in sdLDL-C by the direct method (**Figure 1**).

Furthermore, no significant differences in sdLDL-C concentrations or LDL particle size were observed in patients with type 1 diabetes according to statin therapy, smoking status, sex, or HbA1c levels (≤7.5% vs >7.5%), as summarized in **Table 2**.

**Table 2:** sdLDL concentration and LDL particle size across subgroups in patients with type 1 diabetes.

Variables	Sex		Smoking status		HbA_1c_, %		Statin treatment		
	Male	Female	Smoking	Non-smoking	≤ 7.5	> 7.5	Yes		No
sdLDL-C, mmol/L	0.58 ± 0.26	0.54 ± 0.32	0.55 ± 0.34	0.57 ± 0.25	0.56 ± 0.20	0.57 ± 0.35	0.60 ± 0.28		0.54 ± 0.29
p	0.575		0.739		0.864		0.385		
LDL size, nm	26.39 ± 1.09	26.29 ± 0.99	26.19 ± 1.00	26.43 ± 1.07	26.34 ± 1.07	26.35 ± 1.05	26.42 ± 1.16	26.28 ± 0.95	
p	0.686		0.364		0.952		0.583		

### 3.3 Lipoprotein particle profile by NMR

The complete NMR data are shown in **Table 3**. NMR results showed that the ratio cholesterol (C)/TG of VLDL was significantly lower in patients with type 1 diabetes compared to HC, suggesting increased VLDL particle size. Regarding LDL, and consistent with the biochemical findings, NMR confirmed the reduction in LDL-C levels in patients with type 1 diabetes vs HC, and also revealed lower LDL-TG content in patients with type 1 diabetes compared with HC. Reduced number of total LDL particles (LDL-P) and large, medium and small LDL-P was observed in patients with type 1 diabetes vs HC. The latter result being opposite to that observed using direct sdLDL-C measurement or GGE. Regarding HDL, increased C/TG ratio was observed in type 1 diabetes patients, suggesting larger HDL particles (HDL-P). In agreement, the concentrations of large and medium HDL particles were increased whereas small HDL particles were decreased in type 1 diabetes patients. Accordingly, the ratio small HDL-P/HDL-P was decreased in type 1 diabetes patients compared with HC controls.

**Table 3:** Advanced lipoprotein profile obtained by NMR analysis.

Lipoprotein profile variables	HC (n=24)	Type 1 diabetes (n=69)	p
VLDL-C (mg/dL)	12.78 ± 6.24	12.36 ± 8.11	0.2034
VLDL-TG (mg/dL)	40.09 ± 13.26	51.90 ± 32.44	0.3329
Ratio VLDL-C/TG	0.30 ± 0.06	0.23 ± 0.08	**0.0067**
VLDL-P (nmol/L)	30.90 ± 11.37	37.00 ± 21.03	0.5321
Large VLDL-P (nmol/L)	0.82 ± 0.27	0.97 ± 0.44	0.4222
Medium VLDL-P (nmol/L)	3.98 ± 1.13	4.79 ± 3.78	0.2693
Small VLDL-P (nmol/L)	26.09 ± 1.11	31.23 ± 17.16	0.1887
IDL-C (mg/dL)	8.60 ± 2.78	8.14 ± 3.16	0.4967
IDL-TG (mg/dL)	8.73 ± 2.09	8.82 ± 3.53	0.9916
LDL-C (mg/dL)	133.37 ± 18.67	118.50 ± 14.6	**<0.0001**
LDL-TG (mg/dL)	14.04 ± 2.92	12.74 ± 4.74	**0.0052**
Ratio LDL-C/TG	9.68 ± 1.22	9.88 ± 2.04	0.7944
LDL-P (nmol/L)	1276.4 ± 184.7	1138.5 ± 149.5	**0.0001**
Large LDL-P (nmol/L)	220.8 ± 28.3	205.2 ± 27.6	**0.0137**
Medium LDL-P (nmol/L)	399.9 ± 81.4	329.9 ± 67.7	**<0.0001**
Small LDL-P (nmol/L)	655.7 ± 103.1	603.4 ± 81.9	**0.0071**
Small LDL-P/LDL-P	0.52 ± 0.03	0.53 ± 0.04	0.3441
HDL-C (mg/dL)	59.64 ± 12.23	61.83 ± 12.48	0.2895
HDL-TG (mg/dL)	12.36 ± 2.52	11.59 ± 4.17	0.0775
Ratio HDL-C/TG	5.04 ± 1.60	6.03 ± 2.93	**0.0269**
HDL-P (μmol/L)	28.63 ± 4.86	27.69 ± 5.14	0.7717
Large HDL-P (μmol/L)	0.28 ± 0.04	0.31 ± 0.03	**0.0003**
Medium HDL-P (μmol/L)	10.77 ± 1.92	12.03 ± 2.02	**0.0055**
Small HDL-P (μmol/L)	17.58 ± 3.43	15.34 ± 4.16	**0.0173**
Small HDL-P/HDL-P	0.62 ± 0.04	0.54 ± 0.08	**<0.0001**

HC, Healthy controls; C, Cholesterol; TG, Triglycerides; P, Particles. Data is expressed as mean ± SD. Bold numbers indicate statistically significant differences.

### 3.4 Lipoprotein metabolism regulators

**Figure 2:**
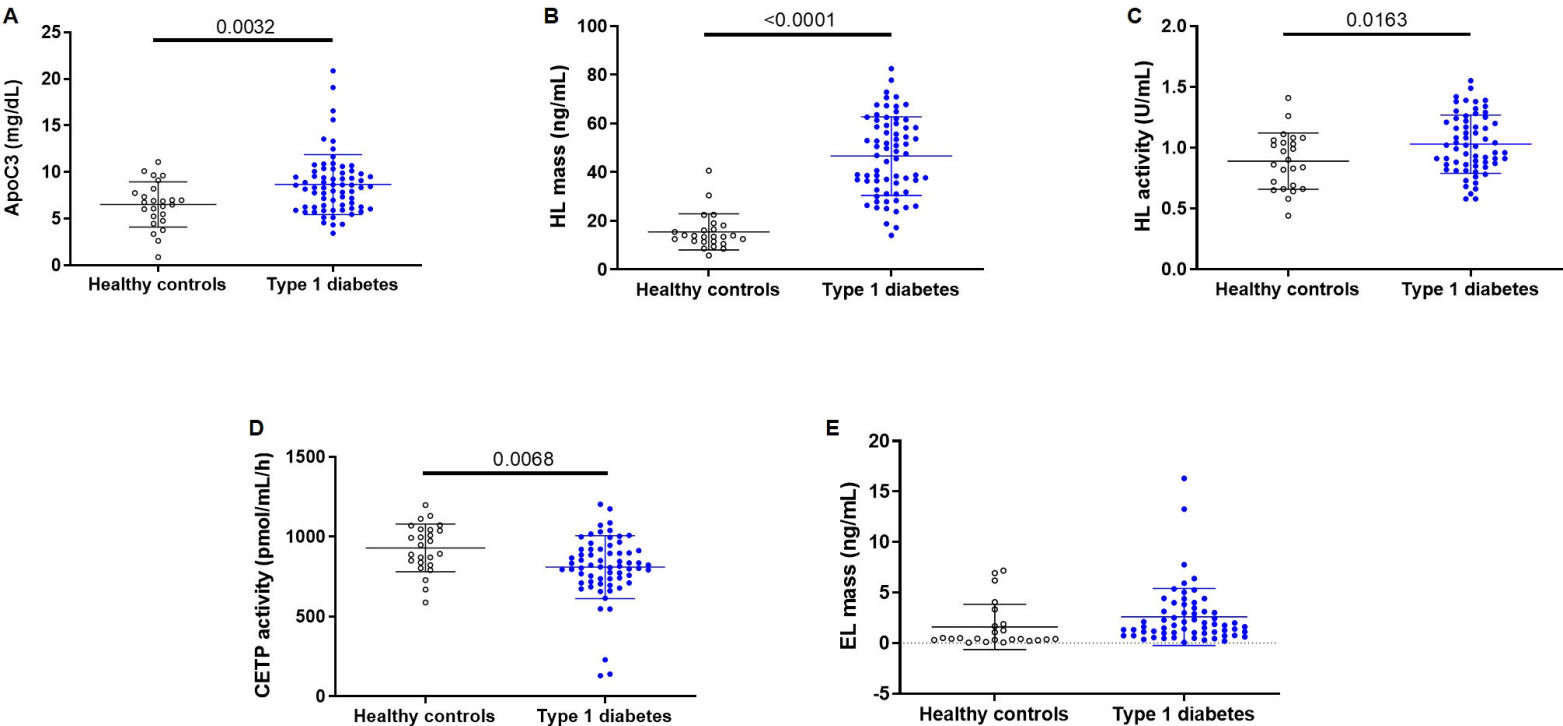
Title: ApoC3 and lipid metabolism enzymes in patients with type 1 diabetes and healthy controls. **(A)** ApoC3 mass, **(B)** HL mass, **(C)** HL activity **(D)**, CETP activity and **(E)** EL mass. Horizontal lines indicate statistical differences.

**Figure 2** shows the concentration or activity of enzymes and factors involved in sdLDL formation. ApoC3 concentrations (8.67 ± 3.22 mg/dL vs 6.53 ± 2.42) and HL mass (46.60 ± 16.12 ng/mL vs 15.45 ± 7.40 ng/mL) and activity (1.03 ± 0.24 U/mL vs 0.89 ± 0.23 U/mL), were significantly higher in type 1 diabetes patients compared with HC (p < 0.05), while CETP activity was decreased (808.8 ± 197.0 pmol/mL/h vs 929.7 ± 149.6 pmol/mL/h; p = 0.0068). EL concentrations were not significantly different between groups (2.59 ± 2.83 ng/mL vs 1.60 ± 2.24 ng/mL; p > 0.05).

### 3.5 Correlation analysis

**Figure 3:**
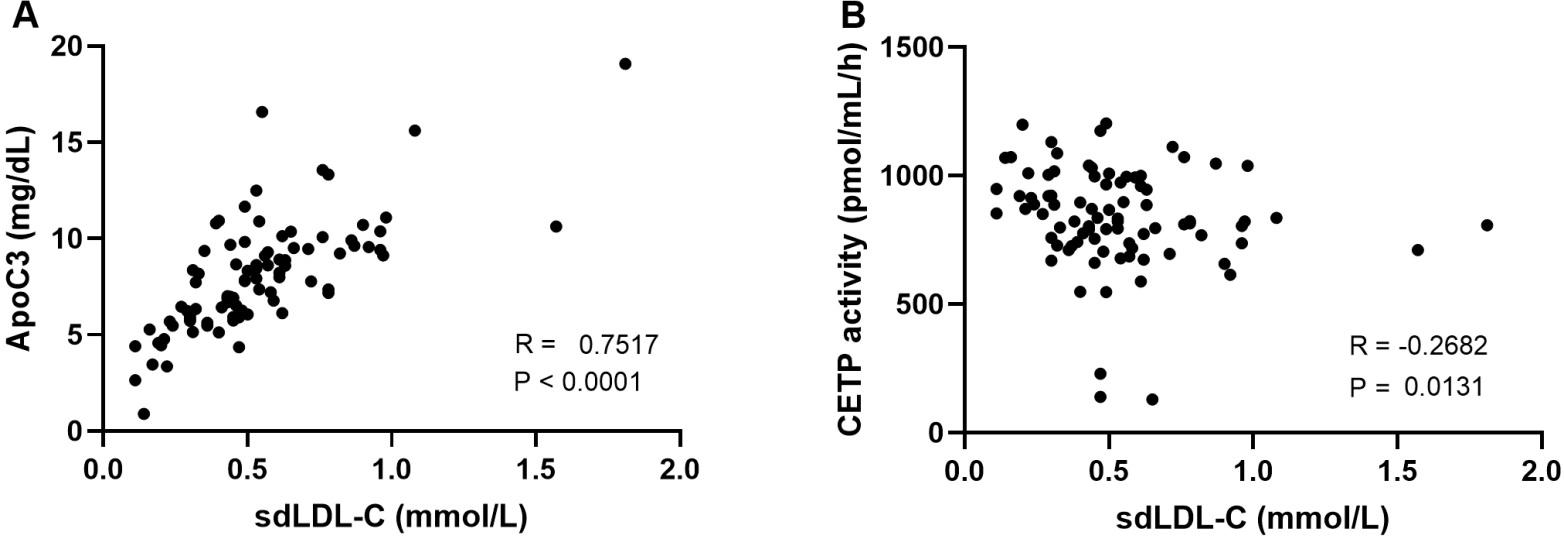
Title: Spearman’s rho correlation test of parameters statistically associated to sdLDL-C. **(A)** apolipoprotein C3 (ApoC3) mass, **(B)** cholesteryl ester transfer protein (CETP) activity.

Correlation analyses revealed a significantly positive correlation between sdLDL-C concentration and ApoC3, and a negative correlation between sdLDL concentration and CETP activity (**Figure 3**). sdLDL showed no significant correlations with HL mass and activity, or EL mass (see Supplementary File **Supplementary Table 1**).

## 4 Discussion

In this study, individuals with type 1 diabetes exhibited higher concentrations of sdLDL-C compared with HC, despite normal TG and HDL-C levels, indicating the presence of a more atherogenic lipoprotein profile. Furthermore, although individuals with type 1 diabetes present lower LDL-C concentrations, we observed a higher sdLDL-C/LDL-C ratio, indicating a relative enrichment of sdLDL particles. These findings are consistent with previous reports (5, 6, 10, 13, 30) and further suggest that conventional lipid panels may underestimate cardiovascular risk in type 1 diabetes. Importantly, our study extends these observations by applying complementary methodologies for sdLDL quantification and by simultaneously evaluating key regulators of lipoprotein metabolism, including ApoC3, HL, CETP, and EL, providing a more comprehensive characterization of sdLDL alterations in this population.

Prior studies have yielded heterogeneous findings regarding sdLDL in type 1 diabetes, largely due to methodological differences and variations in patient characteristics (4, 5, 9–14). Ultracentrifugation and chromatographic approaches generally demonstrate increased sdLDL (5, 10, 13), whereas NMR spectroscopy has often yielded neutral or even opposite results (4, 9, 11, 14). In our cohort, NMR showed fewer small LDL particles in individuals with type 1 diabetes. This apparent discrepancy, likely reflects differences in LDL particles classification across methods, as NMR typically assigns a larger proportion of LDL particles to the “small LDL” fraction (approximately 50%), compared with direct sdLDL-C quantification methods, which usually identify about 10-20% of LDL-C as sdLDL-C. These methodological differences highlight the importance of using complementary analytical approaches for accurate sdLDL characterization.

In type 2 diabetes and metabolic syndrome, sdLDL formation is typically driven by hypertriglyceridemia and insulin resistance. In these conditions, excess TG-rich VLDL particles promote CETP-mediated triglyceride transfer from VLDL to LDL, generating TG-enriched LDL particles that are subsequently hydrolyzed by HL into smaller, denser LDL (3). By contrast, the presence of increased sdLDL in our normotriglyceridemic type 1 diabetes cohort is not metabolically expected. Importantly, these patients had near-normal lipid levels (cholesterol <5.8 mmol/L, triglycerides <2.5 mmol/L), long disease duration, and adequate glycemic control (HbA_1c_ ~7–7.5%), supporting the existence of disease-specific mechanisms driving lipoprotein remodeling independent of classical insulin resistance state.

Consistent with this concept, the HDL profile in our cohort, characterized by larger HDL particles and fewer small HDL particles, further distinguishes the lipoprotein phenotype of type 1 diabetes from the classical insulin-resistant dyslipidemia, where sdLDL enrichment is accompanied by hypertriglyceridemia, reduced HDL-C levels, and smaller HDL particles. This pattern supports a distinct lipoprotein remodeling process in type 1 diabetes, likely related to altered VLDL composition and regulatory protein activity, rather than hypertriglyceridemia.

Several mechanisms may underlie this lipoprotein remodeling observed in type 1 diabetes. Subcutaneous insulin therapy creates a non-physiological portal-to-peripheral insulin gradient, with relatively reduced hepatic insulin exposure and higher systemic insulin levels. This altered insulin distribution may influence hepatic lipid metabolism and promote the secretion of fewer but larger TG-enriched VLDL particles, consistent with the reduced VLDL cholesterol-to-triglyceride ratio observed in our cohort and with previous reports describing altered VLDL composition in type 1 diabetes (31, 32). Importantly, abnormalities in lipoprotein composition may persist despite near-normal glycemic control under conventional subcutaneous insulin therapy (6, 33, 34). In contrast, insulin delivery into the portal circulation has been shown to improve lipoprotein metabolism. Studies using intraperitoneal insulin in type 1 diabetes reported reductions in plasma TG and LDL-C together with increases in HDL-C, as well as normalization of lipoprotein composition with lower triglyceride enrichment of LDL and HDL and reduced prevalence of sdLDL particles. Together, these observations suggest that the route of insulin administration may influence hepatic lipoprotein remodeling and could contribute to sdLDL enrichment in type 1 diabetes (35).

In this context, the reduced CETP activity observed in our cohort contrasting with earlier reports showing increased CETP activity in type 1 diabetes (36), may reflect this altered hepatic insulin exposure. Such altered hepatic insulin exposure may influence VLDL composition and lipoprotein remodeling pathways, potentially explaining the lower CETP activity observed. Lower CETP activity limits the exchange of triglycerides and cholesteryl esters between VLDL and HDL, favoring the persistence of TG-enriched VLDL and larger HDL particles. These particles may subsequently serve as substrates for HL, facilitating the formation of sdLDL (37, 38). Importantly, CETP activity was inversely associated with sdLDL-C concentration, supporting its functional relevance in this process.

In addition, ApoC3 was elevated in our cohort, consistent with prior studies (19–21). This increase may reflect impaired hepatic insulin signaling under subcutaneous insulin therapy as insulin normally represses *APOC3* transcription via FoxO1, whereas the absence of physiological portal insulin delivery attenuates this suppression despite systemic hyperinsulinemia (20, 39). Increased ApoC3 prolongs the residence time of TG-rich lipoproteins by inhibiting lipoprotein lipase (LpL) and delaying hepatic clearance (20), thereby favoring the generation of TG-enriched LDL precursors. HL mass and activity were also increased in our cohort, in agreement with previous studies in type 1 diabetes (40, 41). Although HL did not correlate directly with sdLDL-C levels, its increased activity may facilitate the hydrolysis of TG-enriched LDL precursors into smaller, denser particles. Experimental studies have confirmed that HL facilitates the conversion of TG-rich LDL into sdLDL through surface phospholipid and TG hydrolysis (42).

Taken together, our results suggest that sdLDL-C accumulation in type 1 diabetes arises from a combination of elevated ApoC3, increased HL activity, reduced CETP activity and altered VLDL composition, independently of classical insulin resistance or overt hypertriglyceridemia. In particular, the strong positive association between ApoC3 and sdLDL-C concentration, together with the inverse association with CETP activity, supports a potential contribution of these factors to sdLDL-C formation in this population, with the route of insulin administration representing a possible upstream determinant of these alterations.

The strengths of our study include the simultaneous application of complementary methodologies for sdLDL quantification, the integrated analysis of key lipoprotein regulators, and the focus on a well-characterized cohort with long disease duration and adequate and clinically representative glycemic control. However, several limitations should be considered. The relatively small sample size may limit statistical power and the ability to detect subtle differences or associations. Post-heparin LpL and HL activity measurements were not available, limiting the assessment of lipoprotein remodeling. The cross-sectional design also precludes causal inference between sdLDL-C concentrations, lipoprotein regulations, and clinical outcomes. Furthermore, the study population consisted exclusively of Caucasian individuals from a single tertiary center, which may limit the generalizability of our findings to other ethnic groups or clinical settings. Finally, residual confounding from unmeasured factors such as diet, physical activity, or medication adherence cannot be completely excluded.

## 5 Conclusions

In conclusion, patients with type 1 diabetes exhibit increased sdLDL-C despite near-normal lipid levels, helping to reconcile previously conflicting reports derived from heterogeneous methodological approaches. This phenotype is linked to elevated ApoC3 and HL, reduced CETP activity, and may be influenced by altered VLDL composition. These findings provide mechanistic insights into the residual cardiovascular risk of type 1 diabetes and highlight ApoC3, CETP and HL as potential therapeutic targets. Future studies should determine whether modulation of these pathways can reduce sdLDL burden and ultimately lower CVD risk in this population.

## Data Availability

The original contributions presented in the study are included in the article/**Supplementary Material**. Further inquiries can be directed to the corresponding authors.

## References

[1] Colom C Rull A Sanchez-Quesada JL Pérez A . Cardiovascular disease in type 1 diabetes mellitus: Epidemiology and management of cardiovascular risk. J Clin Med. (2021) 10:1798. doi: 10.3390/jcm10081798. PMID: 33924265 PMC8074744

[2] Lind M Svensson A-M Kosiborod M Gudbjörnsdottir S Pivodic A Wedel H . Glycemic control and excess mortality in type 1 diabetes. N Engl J Med. (2014) 371:1972–82. doi: 10.1056/NEJMoa1408214. PMID: 25409370

[3] O’Brien ST Neylon OM O’Brien T . Dyslipidaemia in type 1 diabetes: Molecular mechanisms and therapeutic opportunities. Biomedicines. (2021) 9:826. doi: 10.3390/biomedicines9070826. PMID: 34356890 PMC8301346

[4] Brugnara L Mallol R Ribalta J Vinaixa M Murillo S Casserras T . Improving assessment of lipoprotein profile in type 1 diabetes by 1H NMR spectroscopy. PloS One. (2015) 10:e0136348. doi: 10.1371/journal.pone.0136348. PMID: 26317989 PMC4552656

[5] Guy J Ogden L Wadwa RP Hamman RF Mayer-Davis EJ Liese AD . Lipid and lipoprotein profiles in youth with and without type 1 diabetes. Diabetes Care. (2009) 32:416–20. doi: 10.2337/dc08-1775. PMID: 19092167 PMC2646019

[6] Pérez A Caixàs A Carreras G Mauricio D Pou J-M Serrat J . Lipoprotein compositional abnormalities in type I diabetes: Effect of improved glycaemic control. Diabetes Res Clin Pract. (1997) 36:83–90. doi: 10.1016/S0168-8227(97)00033-8. PMID: 9229192

[7] Du Z Qin Y . Dyslipidemia and cardiovascular disease: Current knowledge, existing challenges, and new opportunities for management strategies. J Clin Med. (2023) 12:363. doi: 10.3390/jcm12010363. PMID: 36615163 PMC9820834

[8] Packard CJ Boren J Taskinen M-R . Causes and consequences of hypertriglyceridemia. Front Endocrinol (Laus). (2020) 11:252. doi: 10.3389/fendo.2020.00252. PMID: 32477261 PMC7239992

[9] Amor AJ Castelblanco E Hernández M Gimenez M Granado-Casas M Blanco J . Advanced lipoprotein profile disturbances in type 1 diabetes mellitus: A focus on LDL particles. Cardiovasc Diabetol. (2020) 19:126. doi: 10.1186/s12933-020-01099-0. PMID: 32772924 PMC7416413

[10] Maahs DM Hokanson JE Wang H Kinney GL Snell-Bergeon JK East A . Lipoprotein subfraction cholesterol distribution is proatherogenic in women with type 1 diabetes and insulin resistance. Diabetes. (2010) 59:1771–9. doi: 10.2337/db09-1626. PMID: 20393149 PMC2889778

[11] Serés-Noriega T Ortega E Giménez M Perea V Boswell L Mariaca K . Advanced lipoprotein profile identifies atherosclerosis better than conventional lipids in type 1 diabetes at high cardiovascular risk. Nutri Metab Cardiovasc Dis. (2023) 33:1235–44. doi: 10.1016/j.numecd.2023.03.025. PMID: 37088651

[12] Pérez A Wägner AM Carreras G Giménez G Sánchez-Quesada JL Rigla M . Prevalence and phenotypic distribution of dyslipidemia in type 1 diabetes mellitus. Arch Intern Med. (2000) 160:2756. doi: 10.1001/archinte.160.18.2756. PMID: 11025785

[13] Cree-Green M Maahs DM Ferland A Hokanson JE Wang H Pyle L . Lipoprotein subfraction cholesterol distribution is more atherogenic in insulin resistant adolescents with type 1 diabetes. Pediatr Diabetes. (2016) 17:257–65. doi: 10.1111/pedi.12277. PMID: 26080650 PMC4887262

[14] Colhoun HM Otvos JD Rubens MB Taskinen MR Underwood SR Fuller JH . Lipoprotein subclasses and particle sizes and their relationship with coronary artery calcification in men and women with and without type 1 diabetes. Diabetes. (2002) 51:1949–56. doi: 10.2337/diabetes.51.6.1949. PMID: 12031985

[15] Arisaka O Ichikawa G Koyama S Shimura N . Relation between low-density lipoprotein particle size and insulin and diabetes mellitus. J Pediatr. (2009) 155:600. doi: 10.1016/j.jpeds.2009.05.036. PMID: 19773013

[16] Witte DR Taskinen MR Perttunen-Nio H van Tol A Livingstone S Colhoun HM . Study of agreement between LDL size as measured by nuclear magnetic resonance and gradient gel electrophoresis. J Lipid Res. (2004) 45:1069–76. doi: 10.1194/jlr.M300395-JLR200. PMID: 14993238

[17] Ito Y Fujimura M Ohta M Hirano T . Development of a homogeneous assay for measurement of small dense LDL cholesterol. Clin Chem. (2011) 57:57–65. doi: 10.1373/clinchem.2010.149559. PMID: 21051530

[18] Tsai MY Steffen BT Guan W McClelland RL Warnick R McConnell J . New automated assay of small dense low-density lipoprotein cholesterol identifies risk of coronary heart disease. Arterioscler Thromb Vasc Biol. (2014) 34:196–201. doi: 10.1161/ATVBAHA.113.302401. PMID: 24233487 PMC4211254

[19] Pan B-Y Chen C-S Chen F-Y Shen M-Y . Multifaceted role of apolipoprotein C3 in cardiovascular disease risk and metabolic disorder in diabetes. Int J Mol Sci. (2024) 25:12759. doi: 10.3390/ijms252312759. PMID: 39684468 PMC11641554

[20] Valladolid-Acebes I Berggren P-O Juntti-Berggren L . Apolipoprotein CIII is an important piece in the type-1 diabetes jigsaw puzzle. Int J Mol Sci. (2021) 22:932. doi: 10.3390/ijms22020932. PMID: 33477763 PMC7832341

[21] Juntti-Berggren L Refai E Appelskog I Andersson M Imreh G Dekki N . Apolipoprotein CIII promotes Ca ^2+^ -dependent β cell death in type 1 diabetes. Proc Natl Acad Sci. (2004) 101:10090–4. doi: 10.1073/pnas.0403551101. PMID: 15210953 PMC454169

[22] Sibley SD Palmer JP Hirsch IB Brunzell JD . Visceral obesity, hepatic lipase activity, and dyslipidemia in type 1 diabetes. J Clin Endocrinol Metab. (2003) 88:3379–84. doi: 10.1210/jc.2002-021693. PMID: 12843191

[23] Perret B Mabile L Martinez L Tercé F Barbaras R Collet X . Hepatic lipase: Structure/function relationship, synthesis, and regulation. J Lipid Res. (2002) 43:1163–9. doi: 10.1194/jlr.r100020-jlr200, PMID: 12177160

[24] Colhoun HM Scheek LM Rubens MB Van Gent T Underwood SR Fuller JH . Lipid transfer protein activities in type 1 diabetic patients without renal failure and nondiabetic control subjects and their association with coronary artery calcification. Diabetes. (2001) 50:652–9. doi: 10.2337/diabetes.50.3.652. PMID: 11246887

[25] Colom C Viladés D Pérez-Cuellar M Leta R Rivas-Urbina A Carreras G . Associations between epicardial adipose tissue, subclinical atherosclerosis and high-density lipoprotein composition in type 1 diabetes. Cardiovasc Diabetol. (2018) 17:156. doi: 10.1186/s12933-018-0794-9. PMID: 30526614 PMC6284304

[26] ElSayed NA McCoy RG Aleppo G Balapattabi K Beverly EA Briggs Early K . 2. Diagnosis and classification of diabetes: Standards of care in diabetes—2025. Diabetes Care. (2025) 48:S27–49. doi: 10.2337/dc25-S002. PMID: 39651986 PMC11635041

[27] Nichols AV Krauss RM Musliner TA . Nondenaturing polyacrylamide gradient gel electrophoresis. Methods Enzymol. (1986) 128:417–31. doi: 10.1016/0076-6879(86)28084-2. PMID: 3724517

[28] Sánchez-Quesada JL Benítez S Otal C Franco M Blanco-Vaca F Ordóñez-Llanos J . Density distribution of electronegative LDL in normolipemic and hyperlipemic subjects. J Lipid Res. (2002) 43:699–705. doi: 10.1016/s0022-2275(20)30111-5 11971940

[29] Mallol R Amigó N Rodríguez MA Heras M Vinaixa M Plana N . Liposcale: A novel advanced lipoprotein test based on 2D diffusion-ordered 1H NMR spectroscopy. J Lipid Res. (2015) 56:737–46. doi: 10.1194/jlr.D050120. PMID: 25568061 PMC4340320

[30] Bojanin D Vekic J Milenkovic T Vukovic R Zeljkovic A Stefanovic A . Association between proprotein convertase subtilisin/kexin 9 (PCSK9) and lipoprotein subclasses in children with type 1 diabetes mellitus: Effects of glycemic control. Atherosclerosis. (2019) 280:14–20. doi: 10.1016/j.atherosclerosis.2018.11.020. PMID: 30453116

[31] Christ ER Carroll PV Albany E Umpleby AM Lumb PJ Wierzbicki AS . Normal VLDL metabolism despite altered lipoprotein composition in type 1 diabetes mellitus. Clin Endocrinol (Oxf). (2001) 55:777–87. doi: 10.1046/j.1365-2265.2001.01407.x. PMID: 11895220

[32] Llauradó G Amigó N Cano A Ballesta S Albert L Mazarico I . Specific nuclear magnetic resonance lipoprotein subclass profiles and central arterial stiffness in type 1 diabetes mellitus: A case control study. J Clin Med. (2019) 8:1875. doi: 10.3390/jcm8111875. PMID: 31694246 PMC6912486

[33] Bagdade JD Dunn FL . Effects of insulin treatment on lipoprotein composition and function in patients with IDDM. Diabetes. (1992) 41:107–10. doi: 10.2337/diab.41.2.S107. PMID: 1526328

[34] Dunn FL . Plasma lipid and lipoprotein disorders in IDDM. Diabetes. (1992) 41:102–6. doi: 10.2337/diab.41.2.S102. PMID: 1526327

[35] Bagdade JD Dunn FL . Improved lipoprotein surface and core lipid composition following intraperitoneal insulin delivery in insulin-dependent diabetes mellitus. Diabetes Metab. (1996) 22:420–6. 8985650

[36] Bouillet B Gautier T Blache D Pais De Barros JP Duvillard L Petit JM . Glycation of apolipoprotein C1 impairs its CETP inhibitory property: Pathophysiological relevance in patients with type 1 and type 2 diabetes. Diabetes Care. (2014) 37:1148–56. doi: 10.2337/dc13-1467. PMID: 24574346

[37] Berneis KK Krauss RM . Metabolic origins and clinical significance of LDL heterogeneity. J Lipid Res. (2002) 43:1363–79. doi: 10.1194/jlr.R200004-JLR200. PMID: 12235168

[38] Packard CJ Shepherd J . Lipoprotein heterogeneity and apolipoprotein B metabolism. Arterioscler Thromb Vasc Biol. (1997) 17:3542–56. doi: 10.1161/01.ATV.17.12.3542. PMID: 9437204

[39] Altomonte J Cong L Harbaran S Richter A Xu J Meseck M . Foxo1 mediates insulin action on apoC-III and triglyceride metabolism. J Clin Invest. (2004) 114:1493–503. doi: 10.1172/JCI19992. PMID: 15546000 PMC525736

[40] Hughes TA Calderon RM Diaz S Mendez AJ Goldberg RB . Lipoprotein composition in patients with type 1 diabetes mellitus: Impact of lipases and adipokines. J Diabetes Complicat. (2016) 30:657–68. doi: 10.1016/j.jdiacomp.2016.01.018. PMID: 26997169

[41] Calderon RM Diaz S Szeto A Llinas JA Hughes TA Mendez AJ . Elevated lipoprotein lipase activity does not account for the association between adiponectin and HDL in type 1 diabetes. J Clin Endocrinol Metab. (2015) 100:2581–8. doi: 10.1210/jc.2015-1357. PMID: 25942477 PMC8210875

[42] Jansen H Verhoeven AJM Sijbrands EJG . Hepatic lipase. J Lipid Res. (2002) 43:1352–62. doi: 10.1194/jlr.R200008-JLR200. PMID: 12235167

