# Supplementary material for: Altered small dense LDL profiles in long-standing controlled type 1 diabetes
Source: Front Endocrinol (Lausanne). 2026 Apr 7;17:1804987. doi: 10.3389/fendo.2026.1804987 (PMC13096098; doi:10.3389/fendo.2026.1804987)
Supplement: Supplementary file 1 [file DataSheet1.docx]

Supplementary Material

# Supplementary Figures


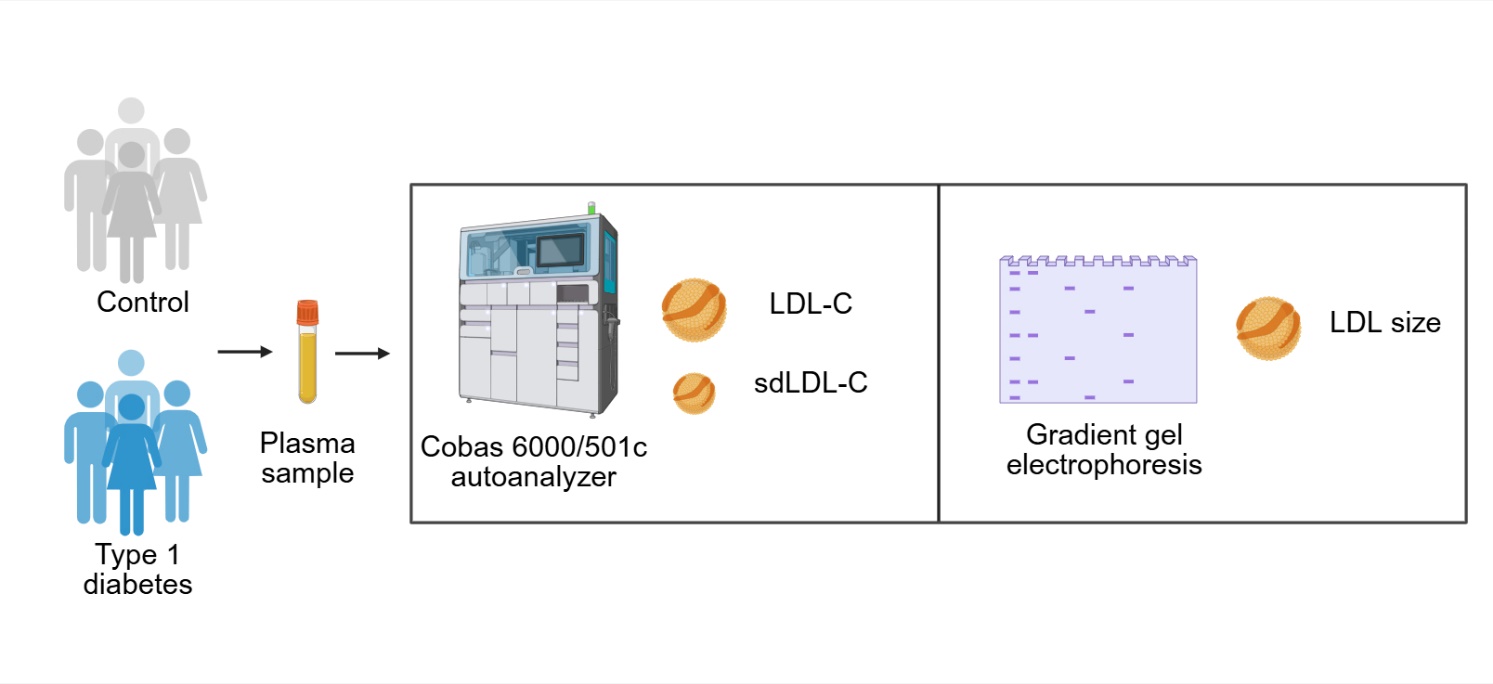


**Figure S1.** Schematic diagram illustrating the analytical methods used for sdLDL-cholesterol (sdLDL-C) quantification by a homogeneous assay adapted for the Cobas 6000/501c platform, and LDL particle size determination by non-denaturing gradient gel electrophoresis (2–16%). Created in BioRender. Rotllan, N. (2025) https://BioRender.com/undefined

**
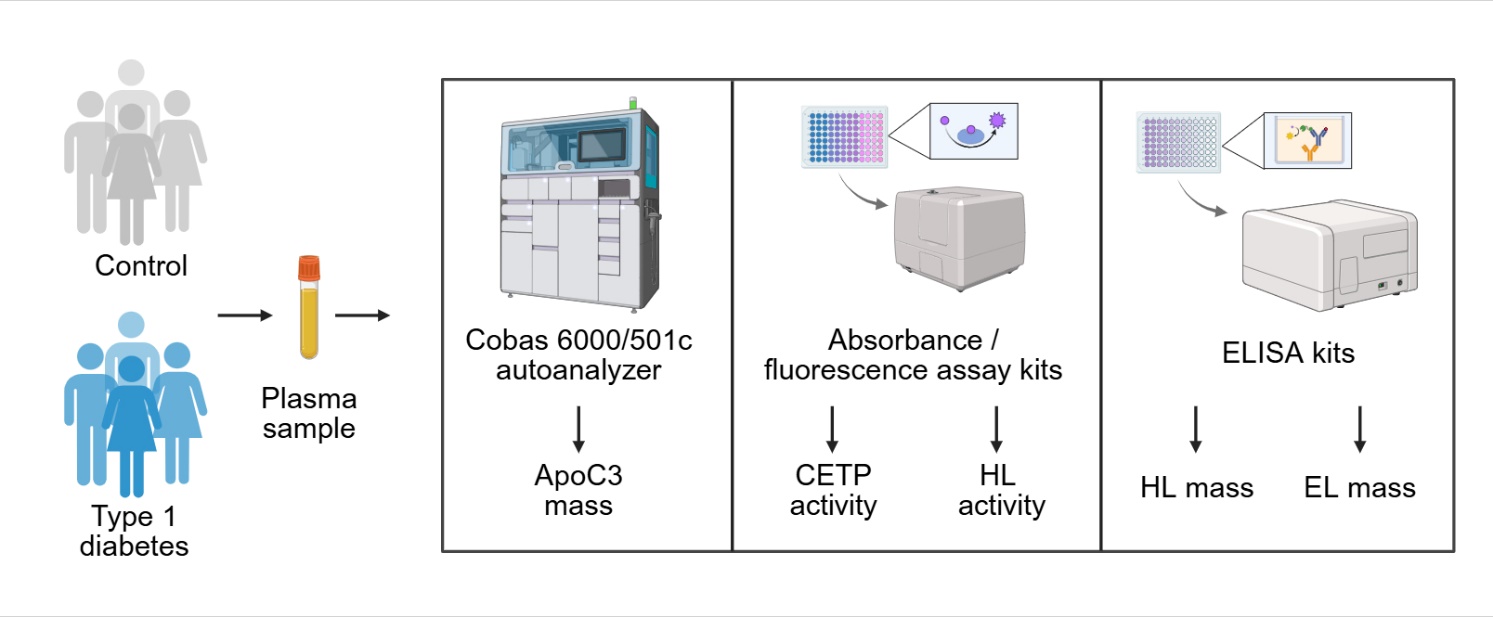
**

**Figure S2.** Schematic diagram illustrating the analytical methods used for ApoC3 quantification by a commercial immunoturbidimetric assay (adapted for the Cobas 6000/501c platform), cholesteryl ester transfer protein (CETP) and hepatic lipase (HL) activities by commercial assays based on fluorescent lipid transfer and α-naphthyl acetate hydrolysis, and HL and endothelial lipase (EL) concentrations by ELISA kits. Created in BioRender. Rotllan, N. (2025) https://BioRender.com/undefined

# Supplementary Table

**Table S1.** Spearman’s rho correlation coefficient analysis of parameters of LDL size, ApoC3 and enzymes involved in LDL metabolism.

| **P** | ApoC3 (mg/dL) | HL mass (ng/mL) | HL activity (U/mL) | EL (ng/mL) | CETP (pmol/mL/h) | sdLDL-C (mmol/L) |
| --- | --- | --- | --- | --- | --- | --- |
| ApoC3 (mg/dL) |  | 0.1038 | 0.2754 | 0.6620 | 0.0266 | 5.95E-18 |
| HL mass (ng/mL) | 0.1038 |  | 0.0186 | 0.0220 | 0.1212 | 0.6183 |
| HL activity (U/mL) | 0.2754 | 0.0186 |  | 0.1277 | 0.0085 | 0.1795 |
| EL (ng/mL) | 0.6620 | 0.0220 | 0.1277 |  | 0.6808 | 0.9521 |
| CETP (pmol/mL/h) | 0.0266 | 0.1212 | 0.0085 | 0.6808 |  | 0.0131 |
| sdLDL-C (mmol/L) | 5.95E-18 | 0.6183 | 0.1795 | 0.9521 | 0.0131 |  |
|  |  |  |  |  |  |  |
|  |  |  |  |  |  |  |
| **R** | ApoC3 (mg/dL) | HL mass (ng/mL) | HL activity (U/mL) | EL (ng/mL) | CETP (pmol/mL/h) | sdLDL-C (mmol/L) |
| ApoC3 (mg/dL) | 1 | 0.1716 | 0.1204 | 0.0478 | -0.2405 | 0.7517 |
| HL mass (ng/mL) | 0.1716 | 1 | 0.2563 | 0.2468 | -0.1694 | 0.0529 |
| HL activity (U/mL) | 0.1204 | 0.2563 | 1 | 0.1686 | -0.2870 | 0.1479 |
| EL (ng/mL) | 0.0478 | 0.2468 | 0.1686 | 1 | -0.0453 | -0.0066 |
| CETP (pmol/mL/h) | -0.2405 | -0.1694 | -0.2870 | -0.0453 | 1 | -0.2682 |
| sdLDL-C (mmol/L) | 0.7517 | 0.0529 | 0.1479 | -0.0066 | -0.2682 | 1 |

Upper panel indicates the correlation coefficient (R) and lower panel indicates statistical significance (P). Statistically significant correlations are indicated in yellow (positive) and in green (negative).

**
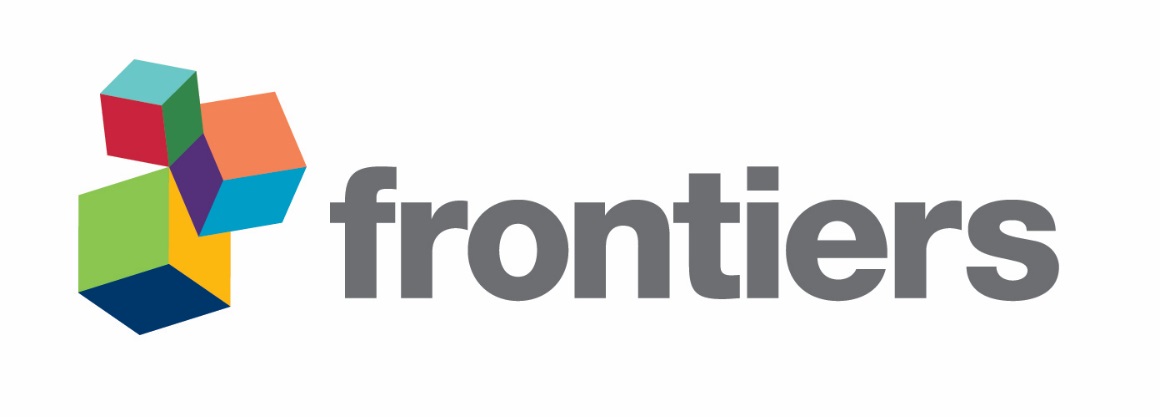
**
